# Resazurin microtitre plate assay and Sensititre® MycoTB for detection of *Mycobacterium tuberculosis* resistance in a high tuberculosis resistance setting

**DOI:** 10.4102/ajlm.v8i1.840

**Published:** 2019-12-13

**Authors:** Prenika Jaglal, Melendhran Pillay, Koleka Mlisana

**Affiliations:** 1 Department of Medical Microbiology, National Health Laboratory Services, School of Laboratory Medicine and Medical Science, University of KwaZulu-Natal, Durban, South Africa; 2 Department of Medical Microbiology/Virology, National Health Laboratory Service, Durban, South Africa

**Keywords:** *Mycobacterium tuberculosis*, agar proportion method, multidrug-resistant tuberculosis, extensively drug-resistant tuberculosis, Sensititre^®^ MycoTB assay, resazurin microtitre plate assay

## Abstract

**Background:**

Rapid diagnosis of drug-resistant *Mycobacterium tuberculosis* is a challenge in low-income countries. Phenotypic drug susceptibility testing using Sensititre^®^ MycoTB assay and the resazurin microtitre plate assay (REMA) are relatively new innovative methods to determine drug susceptibility.

**Objectives:**

This study aimed to determine the performance of the Sensititre and REMA for *M. tuberculosis* drug susceptibility testing in a high-volume tuberculosis reference laboratory.

**Methods:**

A laboratory-based study was performed at the Inkosi Albert Luthuli Central Hospital Tuberculosis Laboratory from January 2014 to June 2015. The Sensititre^®^ MycoTB plate and REMA were compared to the gold standard agar proportion method (APM) using 134 stored isolates.

**Results:**

Agreement between the Sensititre^®^ MycoTB plate and APM was observed with 98% sensitivity, 82% specificity, 94% positive and 93% negative predictive values of the Sensititre^®^ MycoTB assay for the detection of rifampicin resistance and 97%, 96%, 99% and 88% for isoniazid resistance. Good categorical agreement between the REMA and the APM was observed among isolates with 89% sensitivity, 68% specificity, 89% positive and 68% negative predictive value for the detection of rifampicin resistance and 95%, 96%, 99% and 81% for isoniazid resistance. Results for the second-line drugs showed elevated minimum inhibitory concentrations for multidrug-resistant and extensively drug-resistant tuberculosis isolates.

**Conclusion:**

The REMA and Sensititre^®^ MycoTB plate are attractive alternatives to the gold standard APM for the phenotypic detection of *M. tuberculosis* drug resistance.

## Introduction

Multidrug-resistant tuberculosis has been declared a public health crisis by the World Health Organization (WHO).^1^ The global burden of tuberculosis remains enormous with an incidence of 10 million new cases and a mortality of 1.3 million attributed to the disease worldwide in 2017.^1^ The 2018 WHO Global Tuberculosis Report documented new tuberculosis cases, which included an additional 300 000 tuberculosis cases among HIV-positive tuberculosis patients.^1^

South Africa is among the top 20 high-tuberculosis-burden countries worldwide and was previously ranked third following India and China.^1,2^ Multidrug-resistant (MDR) tuberculosis is steadily increasing in South Africa; cases doubled from 7350 in 2007 to 14 000 in 2017.^1,3^ A form of tuberculosis known as extensively drug-resistant (XDR) tuberculosis has been reported in 92 countries. South Africa has reported 73% of the global XDR cases following the historical outbreak of XDR tuberculosis in Tugela Ferry.^4^

The standard tuberculosis treatment regimen lasts for a minimum of 6 months. Treatments regimens for MDR tuberculosis (resistance to isoniazid and rifampicin) and XDR tuberculosis (resistance to any of the injectable drugs including fluoroquinolone, plus MDR tuberculosis),^5,6^ have an extended treatment duration of 18–24 months with harmful second-line anti-tuberculosis drugs. The WHO has recently proposed a 9–12-month shortened regimen duration for MDR tuberculosis, with moxifloxacin replacing gatifloxacin (used in the original Bangladesh regimen).^7,8^

The conventional, culture-based phenotypic method is recognized as the gold standard for confirmation of disease and tuberculosis drug susceptibility testing (DST). The agar proportion method (APM) compares growth of *Mycobacterium* colonies on drug-free and drug-containing mediums, where growth in a particular antibiotic-containing medium determines resistance. It is a low-cost method requiring no special equipment, but with a turnaround time of up to 6 weeks.^9,10^ BACTEC *Mycobacterium* growth indicator tubes (MGIT) 960 liquid culture system (Becton Dickinson) is expensive and prone to contamination but has the advantage of a rapid turnaround time.^2^

The WHO has endorsed the GeneXpert MTB/RIF Ultra system, which is a real-time polymerase chain reaction-based assay for tuberculosis diagnosis and detection of rifampicin resistance, yielding results within 2 hours. The Genotype MTBDR Plus identifies common mutations for rifampicin and isoniazid.^1,11^ Although molecular methods are rapid, they require costly equipment and staff expertise, and cannot detect resistant strains caused by unidentified mutations.^11^

An innovative colorimetric assay has been designed using redox indicators to detect tuberculosis cell viability by a simple colour change. The resazurin microtitre plate assay (REMA) uses resazurin salts in a liquid culture medium.^12,13^ This nontoxic compound incorporates into living cells and is reduced to the fluorescent molecule resorufin via a reduction and oxidation reaction.^14^ Visible colour change of the reagent from blue to pink demonstrates cell viability and therefore drug resistance. This technique has been applied for high throughput screening and determining minimum inhibitory concentrations (MIC) of anti-tuberculosis drugs.^14,15^

Another advance in determining the phenotypic susceptibility of *M. tuberculosis* is the Sensititre^®^ MycoTB (MycoTB TREK Diagnostics) plate method. The Sensititre^®^ MycoTB plate method is a novel 96-well microtitre plate broth microdilution DST method incorporating drug concentrations of 12 anti-tuberculosis drugs for MIC determination.^16^ The drugs included consist of both first-line (rifampicin, isoniazid and ethambutol) as well as second-line drugs (moxifloxacin, ofloxacin, para-aminosalicylic acid, rifabutin, streptomycin, amikacin, cycloserine, ethionamide and kanamycin) existing as lyophilised forms in microtitre wells. A 7–21-day period of incubation is needed to observe culture growth noted as turbidity or cellular deposits at the base of a well.^14,16^

The aim of this study was to determine the performance of the Sensititre^®^ MycoTB plate and REMA as potential tools for tuberculosis DST in a high tuberculosis-burden reference laboratory. This entailed comparison of the REMA and Sensititre assay using the APM as a gold standard in order to evaluate turnaround times, sensitivity, specificity, and positive and negative predictive values.

## Methods

### Ethical considerations

The Biomedical Ethics Research Committee (University of KwaZulu-Natal) granted ethical approval for the use of stored study isolates (reference number BE 268/12).

### Processing and culture of sputum specimens

Previously stored *M. tuberculosis* strains isolated from sputum samples received at the Tuberculosis Laboratory based at the Inkosi Albert Luthuli Central Hospital, Durban, South Africa, from January 2014 to June 2015 were used in this study. Briefly, sputum samples were digested and decontaminated using the N-acetyl-L-cysteine–NaOH-sodium citrate (NALC–NaOH, 2% NaOH final concentration) method and cultured in mycobacterium growth indicator tubes (MGIT). Positive cultures were confirmed for the presence of *M. tuberculosis* using the Ziehl Neelsen or MPT64 antigen assay (SD Bioline, Gyeonggi-do, South Korea). MDR and XDR *M. tuberculosis* isolates were routinely stored in the laboratory. One hundred and fifty stored *M. tuberculosis* isolates were subcultured onto Middlebrook 7H11 agar and incubated at 37 °C for 21 days. A total of 134 isolates had confluent growth and were used in the study.

### Drug susceptibility testing by the agar proportion method

Drug susceptibility of tuberculosis-positive cultures was determined using the indirect APM on Middlebrook 7H10 agar^2,16^ for first- and second-line anti-tuberculosis drugs (rifampicin, isoniazid, kanamycin, moxifloxacin and capreomycin). One hundred microlitres from a positive MGIT was inoculated into each quadrant of the Middlebrook 7H10 DST agar plate and incubated for 21 days. Growth of more than 1% on the antibiotic-containing quadrant when compared to the antibiotic-free growth control was regarded as resistant to the corresponding antibiotic. A susceptible growth control isolate, H37Rv (American Type Culture Collection, 25618), was used in the study.

### Resazurin microtitre plate assay

The susceptibility of MDR, XDR and sensitive tuberculosis isolates were evaluated against first- and second-line anti-tuberculosis drugs by the colorimetric REMA method for the 134 isolates. One hundred microlitres of Middlebrook 7H9 (M7H9) broth was aseptically prepared and dispensed carefully into each of the wells of a flat-bottomed, 96-well microtitre plate with lid (Lasec, Midrand, South Africa). The anti-tuberculosis drugs that were tested using the REMA method included rifampicin, isoniazid, kanamycin, moxifloxacin and capreomycin.

Working solutions of the drugs were initially prepared (four times the final concentration) in M7H9 broth supplemented with 0.5% glycerol, 0.1% Casitone and 10% OADC (oleic acid, albumin, dextrose and catalase). One hundred microlitres of the working drug concentrations (isoniazid 1.0 *µ*g/mL, rifampicin 8.0 *µ*g/mL, kanamycin 10.0 *µ*g/mL, capreomycin 8.0 *µ*g/mL and moxifloxacin 2.0 *µ*g/mL) were added to the wells containing Middlebrook 7H9 broth. The anti-tuberculosis drugs were then further serially diluted twofold to a final concentration consisting of isoniazid (0.03 *µ*g/mL), rifampicin (0.25 *µ*g/mL), kanamycin (2.5 *µ*g/mL), capreomycin (1.0 *µ*g/mL) and moxifloxacin (0.06 *µ*g/mL).

An inoculum turbidity of McFarland standard number 1 was prepared from Middlebrook 7H11 (M7H11) agar, diluted in M7H9 (1:10) broth and thereafter added (100 *µ*L) to each of the drug-free and drug-containing wells.^10,12^ A sterile control and a growth control for each isolate were also included. To prevent evaporation during incubation, sterile M7H9 broth was added to all perimeter wells. The plate was incubated at 37 °C after being sealed in a plastic bag. A working solution of resazurin salt (30 *µ*L of 0.02% concentration) was inoculated after 8 days of incubation into each microtitre well.^10^ After overnight incubation, plates were then read the next day, a total of 9 days turnaround time for results interpretation. A colour change from blue to pink denoted a positive reaction (reduction of resazurin to resorufin) confirming drug resistance due to *M. tuberculosis* cell viability.^10^

### Sensititre^®^ MycoTB assay

Setting up the microtitre plates and their interpretation were performed according to manufacturer’s instructions. Colonies from a culture plate (M7H11) were emulsified in a glass tube containing saline, tween and glass beads until a 0.5 McFarland standard was obtained. One hundred microlitres of the suspension was inoculated into each well. The plates were then incubated at 37 °C. Drug concentrations present in each plate were: 4 *µ*g/mL isoniazid, 16 *µ*g/mL rifampicin, 32 *µ*g/mL ethambutol, 40 *µ*g/mL ethionamide, 40 *µ*g/mL kanamycin, 32 *µ*g/mL ofloxacin, 64 *µ*g/mL para-aminosalicyclic acid, 16 *µ*g/mL rifabutin, 32 *µ*g/mL streptomycin, 256 *µ*g/mL cycloserine, 16 *µ*g/mL amikacin and 8.0 *µ*g/mL moxifloixacin. Observations of the plates were made from day 7 to 10 for the presence of turbidity or cellular material confirming growth of *M. tuberculosis* and therefore resistance.^14,16^ Time to results was calculated as the number of days from plate inoculation (day 0) to plate reading with visible growth (day 7–10).

### Interpretation of results

Currently, there are no MIC-interpretative breakpoints for the broth microdilution assays when testing *M. tuberculosis* isolates on Sensititre plates; accordingly, the endpoint was determined as the first well that did not contain any growth. These antimicrobial drug MIC results were recorded on a data sheet per isolate tested. The reproducibility of Sensititre^®^ MycoTB assay by duplicate testing of the isolates was not performed for conditional (or categorical) agreement between the Sensititre^®^ MycoTB method and APM on initial testing. In this study, for interpretation of the Sensititre data, true resistance by Sensititre^®^ MycoTB was established by comparing the Sensititre MIC to the APM critical concentration. This means that an isolate was considered resistant if the Sensititre MIC was greater than the APM critical concentration and therefore truly susceptible if it was less than or equal to the APM critical concentration. For the REMA method, resistance to each drug was determined when there was growth in wells above the APM critical concentrations of 0.25 *µ*g/mL (isoniazid), 1.0 *µ*g/mL (rifampicin), 2.0 *µ*g/mL (moxifloxacin), 4.0 *µ*g/mL (capreomycin) and 5.0 *µ*g/mL (kanamycin).^18^ REMA plates were therefore interpreted categorically based on the calorimetric reaction. The REMA and Sensititre plates were interpreted by two readers who were blinded to the APM results. The two readers were in agreement with all plates read and therefore there was no discordance in interpretation.

## Results

The 134 isolates utilised comprised clinically derived XDR (*n* = 65), MDR (*n* = 28), pre-XDR (*n* = 3), isoniazid mono-resistant (*n* = 15), rifampicin mono-resistant (*n* = 4) and susceptible (*n* = 19) according to the gold standard. The REMA and Sensititre^®^ MycoTB results for all 134 (100%) clinical isolates were tested and interpreted. The time to results for the Sensititre^®^ MycoTB method was as early as 7 days, with more reliable results being produced within 10 days.

**FIGURE 1:**
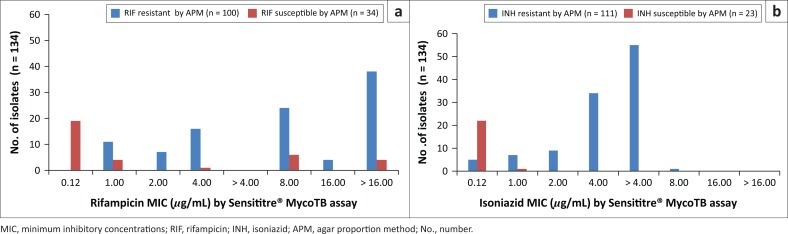
Distribution of isolates by minimum inhibitory concentration for isoniazid and rifampicin, Inkosi Albert Luthuli Central Hospital, Durban, South Africa, January 2014 – June 2015. (a) Distribution of RIF-resistant and -susceptible isolates as determined by APM according to their MIC as determined by the Sensititre® MycoTB assay. (b) Distribution of INH-resistant and -susceptible isolates as determined by APM according to their MIC as determined by the Sensititre® MycoTB assay.

Of the 100 rifampicin-resistant isolates by APM, 98 were resistant by Sensititre assay. Of these 98 isolates, 9 had an MIC of 1 *µ*g/mL, 7 had an MIC of 2 *µ*g/mL, 16 had an MIC of 4 *µ*g/mL, 24 had an MIC of 8 *µ*g/mL, 4 had an MIC of 16 *µ*g/mL and 38 had an MIC above 16 *µ*g/mL. Refer to Figure 1 for rifampicin MIC distributions using the Sensititre^®^ MycoTB.

Of the 16 isolates that were rifampicin-resistant by the Sensititre^®^ MycoTB having MIC of 4 *µ*g/mL, 75% (12/16) were XDR, 12.5% (2/16) MDR and 12.5% (2/16) mono-resistant to rifampicin by the APM. Of the 24 isolates that were resistant to rifampicin by the Sensititre^®^ MycoTB with MIC of 8 *µ*g/mL, 67% (16/24) were XDR, 8% (2/24) pre-XDR and 25% (6/24) were MDR by the APM.

The REMA correctly detected rifampicin resistance in all seven isolates with an MIC of 2 *µ*g/mL by the Sensititre^®^ MycoTB (43% [3/7] XDR, 43% [3/7] MDR and 14% [1/7] mono-resistant to rifampicin by APM) with an MIC of 2 *µ*g/mL by the Sensititre^®^ MycoTB. The REMA confirmed rifampicin resistance in all of the 38 isolates with an MIC above 16 *μ*g/mL by the Sensititre^®^ MycoTB (68% [26/38] XDR and 32% [12/38] MDR) by APM. The overall sensitivity, specificity, positive and negative predictive values of the Sensititre^®^ MycoTB plate for rifampicin-resistance detection were found to be 98%, 82%, 94% and 93% (Table 1). The REMA displayed a sensitivity, specificity, positive and negative predictive values of 89%, 68%, 89% and 68% for the detection of rifampicin resistance (Table 2).

**TABLE 1:** Comparative performance of the Sensititre® MycoTB assay and agar proportion method on multidrug-resistant and extensively drug-resistant tuberculosis isolates, Inkosi Albert Luthuli Central Hospital, Durban, South Africa, January 2014 – June 2015.

Antimicrobial drug	Critical drug concentration of APM (*µ*g/mL)	Sensititre® MycoTB result	APM result (resistant isolates)	APM result (susceptible isolates)	Sensitivity (%)	Specificity (%)	Positive predictive value	Negative predictive value	Accuracy
Isoniazid	0.2	Resistant	108	1	97	96	99	88	97
	-	Susceptible	3	22	-	-	-	-	-
Rifampicin	1	Resistant	98	6	98	82	94	93	94
	-	Susceptible	2	28	-	-	-	-	-
Ofloxacin	2	Resistant	67	2	100	97	97	100	98
	-	Susceptible	0	65	-	-	-	-	-
Moxifloxacin	2	Resistant	21	1	91	98	91	96	96
	-	Susceptible	2	54	-	-	-	-	-
Kanamycin	5	Resistant	59	10	92	85	86	92	87
	-	Susceptible	5	58	-	-	-	-	-

APM, agar proportion method.

**TABLE 2:** Comparative performance of the resazurin microtitre plate assay and agar proportion method on multidrug-resistant and extensively drug-resistant tuberculosis isolates, Inkosi Albert Luthuli Central Hospital, Durban, South Africa, January 2014 – June 2015.

Antimicrobial drug	Critical drug concentration of APM (*µ*g/mL)	REMA result	APM result (resistant isolates)	APM result (susceptible isolates)	Sensitivity (%)	Specificity (%)	Positive predictive value	Negative predictive value	Accuracy
Isoniazid	0.2	Resistant	106	1	95	96	99	81	96
	-	Susceptible	5	22	-	-	-	-	-
Rifampicin	1	Resistant	89	11	89	68	89	68	84
	-	Susceptible	11	23	-	-	-	-	-
Capreomycin	4	Resistant	62	2	93	97	97	93	95
	-	Susceptible	5	65	-	-	-	-	-
Moxifloxacin	2	Resistant	21	12	91	78	64	96	82
	-	Susceptible	2	43	-	-	-	-	-
Kanamycin	5	Resistant	53	4	83	94	93	85	87
	-	Susceptible	11	64	-	-	-	-	-

APM, agar proportion method; REMA, resazurin microtitre plate assay.

Of the 111 isoniazid-resistant isolates by APM, 108 were resistant by the Sensititre assay. Of these 108 isolates, 7 had an MIC of 1 *µ*g/mL, 9 had an MIC of 2 *µ*g/mL, 39 had an MIC of 4 *µ*g/mL, 52 had an MIC of greater than 4 *µ*g/mL and 1 had an MIC above 8 *µ*g/mL. The seven isolates that were isoniazid-resistant by the Sensititre^®^ MycoTB with an MIC of 1 *µ*g/ml (72% MDR and 28% mono-resistant to isoniazid by APM) were also confirmed resistant by the REMA assay. Nine isolates resistant to isoniazid by the Sensititre^®^ MycoTB with an MIC of 2 *µ*g/mL comprised clinically-derived XDR (*n* = 6), MDR (*n* = 2) and pre-XDR (*n* = 1). Resistance to isoniazid by the REMA method was noted in all 9 isolates. Of the 52 isoniazid-resistant isolates with an MIC of over 4 *µ*g/mL by the Sensititre^®^ MycoTB (Figure 1), 25 were XDR, 23 MDR and 4 were mono-resistant to isoniazid by the APM. The REMA assay confirmed isoniazid resistance in 51 of the 52 (98%) isolates. Refer to Figure 1 for isoniazid MIC distributions using the Sensititre^®^ MycoTB. The Sensititre^®^ MycoTB assay sensitivity, specificity, positive and negative predictive values for the detection of isoniazid resistance were found to be 97%, 96%, 99% and 88% (Table 1). The REMA showed sensitivity, specificity, positive and negative predictive values of 95%, 96%, 99% and 81% for the detection of isoniazid resistance (Table 2).

Sensititre^®^ MycoTB testing for the detection of resistance among the MDR and XDR isolates for rifampicin, isoniazid, ofloxacin and kanamycin correlated well with the APM (Table 1). Discrepancies between APM and REMA were observed with moxifloxacin susceptible isolates (APM) where 12 isolates were falsely designated as resistant by REMA (Table 2). The overall sensitivity, specificity, positive and negative predictive values of the Sensititre^®^ MycoTB plate for moxifloxacin-resistance detection were found to be 91%, 98%, 91% and 96% (Table 1). The REMA assay showed sensitivity, specificity, positive and negative predictive values of 91%, 78%, 64% and 96% for the detection of moxifloxacin resistance (Table 2).

The overall sensitivity, specificity, positive and negative predictive values of the Sensititre^®^ MycoTB assay for ofloxacin resistance detection were found to be 100%, 97%, 97% and 100% (Table 1). The sensitivity of detection for ofloxacin resistance was not assessed by the REMA as the drug could not be procured due to limited funds. The overall sensitivity, specificity, positive and negative predictive values of the Sensititre^®^ MycoTB plate for kanamycin resistance detection were found to be 92%, 85%, 86% and 92% (Table 1). The REMA assay showed sensitivity, specificity, positive and negative predictive values of 83%, 94%, 93% and 85% for kanamycin resistance detection (Table 2). Refer to Figure 3 for kanamycin MIC distributions using the Sensititre^®^ MycoTB. Good accuracy with regard to resistance to the first-line anti-tuberculosis drugs was observed with the Sensititre method as compared to the REMA (Tables 1 and 2).

**FIGURE 2:**
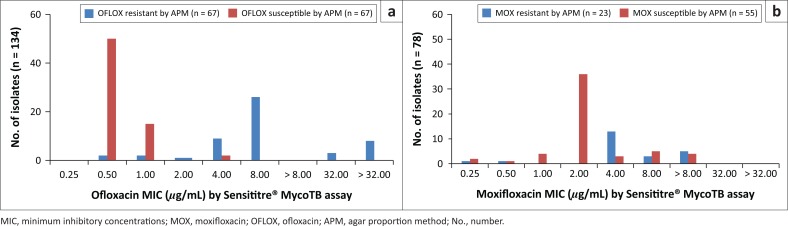
Distribution of isolates by minimum inhibitory concentration for ofloxacin and moxifloxacin, Inkosi Albert Luthuli Central Hospital, Durban, South Africa, January 2014 – June 2015. (a) Distribution of OFLOX-resistant and -susceptible isolates as determined by APM according to their MIC as determined by the Sensititre® MycoTB assay. (b) Distribution of MOX-resistant and - susceptible isolates as determined by APM according to their MIC as determined by the Sensititre® MycoTB assay.

**FIGURE 3:**
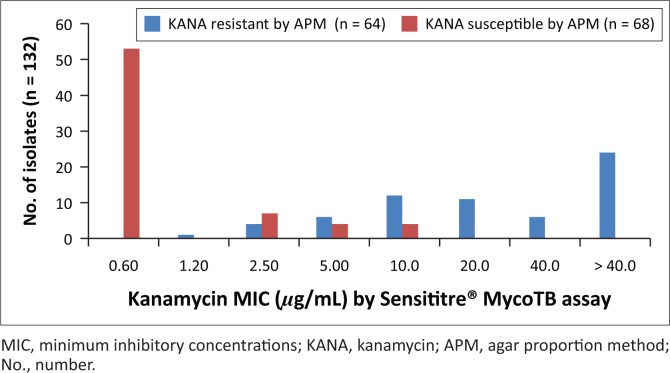
Distribution of isolates by minimum inhibitory concentration for kanamycin, Inkosi Albert Luthuli Central Hospital, Durban, South Africa, January 2014 – June 2015. Distribution of KANA-resistant and -susceptible isolates as determined by APM according to their MIC as determined by the Sensititre® MycoTB assay.

The performance of the REMA for the detection of capreomycin resistance was determined. All of the 62 capreomycin-resistant isolates by the REMA were confirmed to be XDR tuberculosis by the APM (Table 2). Capreomycin was not part of the drug panel included in the Sensititre^®^ MycoTB assay, therefore its performance could not be assessed.

**FIGURE 4:**
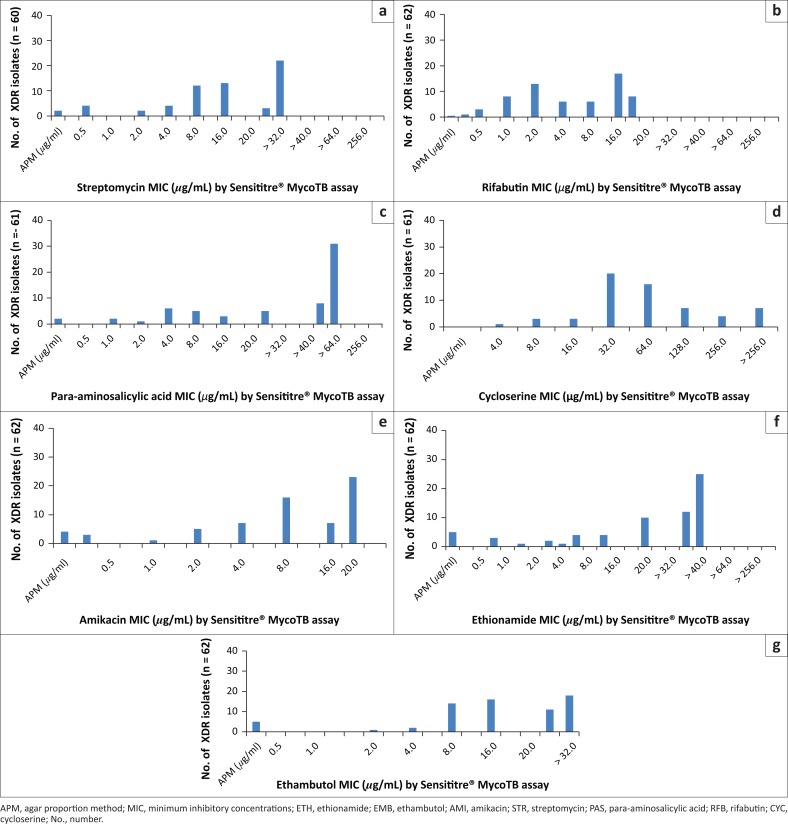
Distribution of minimum inhibitory concentrations (*µ*g/mL) for additional first- and second-line antibiotics using the Sensititre plate method for clinical, extensively drug-resistant tuberculosis isolates, Inkosi Albert Luthuli Central Hospital, Durban, South Africa, January 2014 – June 2015. (a) STR (60 isolates), (b) RFB (62 isolates), (c) PAS (61 isolates), (d) CYC (61 isolates), (e) AMI (62 isolates), (f) ETH (62 isolates) and (g) EMB (62 isolates).

**FIGURE 5:**
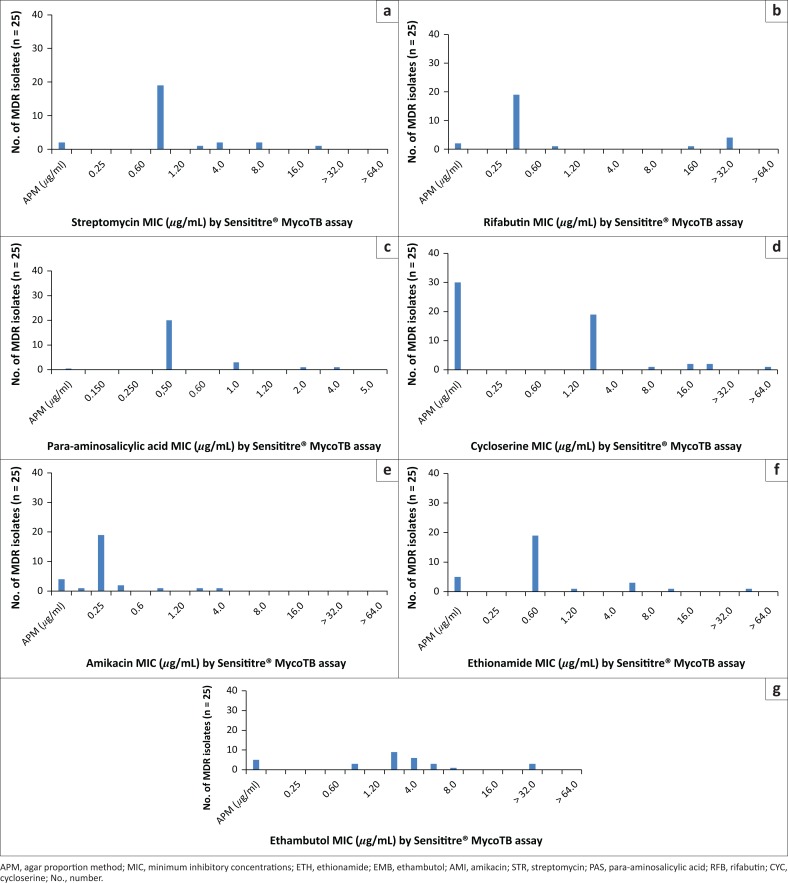
Distribution of minimum inhibitory concentrations (*µ*g/mL) for additional first- and second-line antibiotics using the Sensititre plate method for clinical multidrug-resistant tuberculosis isolates. (a) STR (25 isolates), (b) RFB (25 isolates), (c) PAS (25 isolates), (d) CYC (25 isolates), (e) AMI (25 isolates), (f) ETH (25 isolates) and (g) EMB (25 isolates), Inkosi Albert Luthuli Central Hospital, Durban, South Africa, January 2014 – June 2015.

The levels of resistance among the MDR and XDR tuberculosis isolates to the additional first- and second-line antibiotics were further assessed using the Sensititre^®^ MycoTB assay using established critical concentrations (Figures 4 and 5). Resistance to rifabutin was observed in 93.5% (58/62) of XDR tuberculosis isolates with the majority 27% (17/62) showing an MIC of 16 *µ*g/mL (Figure 4) at a critical concentration of 0.5 *µ*g/mL. In contrast to this, 80% (20/25) of MDR isolates were confirmed to be susceptible to rifabutin with an MIC of 0.5 *µ*g/mL (Figure 5).

Most (58/61, 95%) of the XDR isolates displayed resistance to para-aminosalicyclic acid (Figure 4). At an MIC of 1 *µ*g/mL, 76% (19/25) of MDR isolates displayed susceptibility to para-aminosalicyclic acid (Figure 5). Resistance to amikacin was observed in 46 of the 62 (74%) XDR isolates (Figure 4). Most (59/62, 95%) XDR isolates were resistant to ethambutol. Conversely, 91% (21/23) MDR isolates were susceptible to ethambutol (Figure 5).

Resistance to amikacin was observed in 46 of the 62 (74%) XDR isolates; the majority (*n* = 23) of amikacin-resistant isolates had an MIC of greater than 16 *µ*g/mL (Figure 4). In contrast to this, all of the 25 MDR isolates tested were susceptible to amikacin with a large proportion 76% (19/25) showing an MIC of 0.25 *µ*g/mL (Figure 5). More than half (37/62, 60%) of XDR isolates were resistant to ethionamide with an MIC of 40 *µ*g/mL or more (Figure 4). Among the ethionamide susceptible isolates, 92% (23/25) were MDR with 83% (19/23) showing an MIC of 0.6 *µ*g/mL (Figure 5).

Resistance to streptomycin was observed in 90% (54/60) of XDR isolates with a large proportion of the streptomycin-resistant isolates, 40% (22/54), showing an MIC of more than 32 *µ*g/mL (Figure 4). A susceptible streptomycin MIC of 0.5 *µ*g/mL was observed in 19 of the 25 MDR isolates (Figure 5). Most XDR isolates (54/61, 80%) and 12% (3/25) of MDR isolates had MICs for cycloserine of 32 *µ*g/mL or more (critical concentration of 30 *µ*g/mL; resistant by APM) (Figures 4 and 5).

## Discussion

The objective of tuberculosis DST is to determine resistant strains that are prognostic of treatment failure and relapse. The Sensititre^®^ MycoTB assay provides a faster method of testing first- and second-line tuberculosis drugs and displayed 99.3% concordance with the agar proportion method in prior studies.^19^

In our study, the percentage agreements between Sensititre plate and APM for resistant isoniazid (97%), rifampicin (98%), moxifloxacin (91%) and ofloxacin (100%) were found in similar studies; however, kanamycin had the lowest categorical agreement with the APM (92%).^19^ Discrepant moxifloxacin (APM susceptible) isolates could be due to the subjectivity of the microtitre plate reading. A discrepant analysis would be useful to clarify discordant results, for example, repeat testing of isolates and ruling out technical errors. According to the WHO, current critical concentrations of all anti-tuberculosis drugs will be reviewed.^1^ As phenotypic DST is required for the determination of moxifloxacin susceptibility (WHO recommendation due to poor concordance of Genotype MTBDRsl with APM), this might prove problematic if the Sensititre^®^ MycoTB assay is used.^1^ The distribution of MIC with regard to second-line tuberculosis antimicrobials among MDR and XDR isolates was clearly evident as rising MIC values with the increasingly MDR strains. Resistance to capreomycin has been documented in KwaZulu-Natal in approximately 90% of XDR isolates of newly diagnosed individuals.^17,19^ Both ethambutol and pyrazinamide resistance has been reported as exceeding 60%, the first-line drugs composing the Bangladesh regimen, as described in previous studies.^8^

The Sensititre^®^ MycoTB plate rapidly produced results in comparison to the APM, however, it produced 44% (15/34) false rifampicin-resistant isolates (sensitive by APM). The treatment implication would include patients who were mismanaged with prolonged, toxic second-line therapy in an MDR facility.

The REMA assay had a relatively short turnaround time of 9 days.^21^ Delays in results retrieval and therefore suitable treatment options for resistant tuberculosis may result in the further selection of resistant *M. tuberculosis*, morbidity and mortality in patients afflicted with the disease. Advantages of the REMA format are that it is faster, low cost, easy to interpret and does not need special equipment.^21^ A disadvantage of this method is the potential for aerosolisation since the plates utilise a liquid medium resulting in a biosafety hazard.^15^ In our study, REMA proved to be labour-intensive, requiring individual drug and dye preparation, as well as tedious microtitre plate inoculation. The current study showed the sensitivity and specificity of the REMA are comparable to that of the APM for isoniazid, rifampicin, capreomycin, moxifloxacin and kanamycin to be comparable to previous studies.^21^ Agreement between REMA and the APM was over 90% for resistance testing among MDR and XDR isolates (isoniazid 95%, capreomycin 93%, moxifloxacin 91%).

### Limitations

A limitation with the APM is that the test provides single set critical concentrations and not clear-cut MIC results for each drug in comparison to the Sensititre^®^ MycoTB plate method. Minimum inhibitory concentration values obtained from using the Sensititre^®^ MycoTB plate could possibly be a prospective guide to establishing definite MIC breakpoint values for anti-tuberculosis drugs in the future as there are no interpretive MIC breakpoints that correlate with critical concentrations.

A lack of APM results for most of the second-line antimicrobials meant that the performance of the Sensititre^®^ MycoTB method in conjunction with the APM could only be calculated and assessed against a limited number of antimicrobials. Results for second-line drugs did show elevated MICs for MDR and XDR isolates with the majority of XDR isolates being resistant.

### Conclusion

The Sensititre^®^ MycoTB assay is a desirable alternative method compared to the APM for tuberculosis DST. Simultaneous first- and second-line antimicrobial testing eradicates the need to set up and maintain antimicrobial drug solutions (REMA). The REMA is ideal for use in resource-poor settings due to its low cost and lack of instrumentation.^12^ In comparison to the APM, which has a short shelf life, the Sensititre^®^ MycoTB MIC plate can last for up to 2 years at room temperature. The Sensititre plate may be used together with rapid molecular tests immediately targeting first- and second-line drug testing in scenarios of MDR and XDR tuberculosis.^18^

Little information is available to date on a worldwide, regional and local scale on the use of the Sensititre plate. The comparative performance of Sensititre^®^ MycoTB assay to APM did show much discordance in this study and therefore cannot be recommended as a replacement of the current gold standard. In future, more studies will have to be performed to determine anti-tuberculous drug MIC interpretive breakpoints using wild type and non-wild type isolates, as well as discordant analysis, in order to increase its use in high-burden areas both locally and nationally, especially in the province of KwaZulu-Natal.
